# Host–Parasite Interactions in *Toxoplasma gondii*-Infected Cells: Roles of Mitochondria, Microtubules, and the Parasitophorous Vacuole

**DOI:** 10.3390/ijms252413459

**Published:** 2024-12-16

**Authors:** Sailen Barik, Joel Andrews

**Affiliations:** Department of Biochemistry and Molecular Biology, Frederick P. Whiddon College of Medicine, Mobile, AL 36688, USA; jandrews@southalabama.edu

**Keywords:** *Toxoplasma*, mitochondria, energy metabolism, protozoa, Apicomplexa, microtubule

## Abstract

An intracellular protozoan, the Apicomplexan parasite *Toxoplasma gondii* (*T. gondii*) infects nucleated cells, in which it triggers the formation of a specialized membrane-confined cytoplasmic vacuole, named the parasitophorous vacuole (PV). One of the most prominent events in the parasite’s intracellular life is the congregation of the host cell mitochondria around the PV. However, the significance of this event has remained largely unsolved since the parasite itself possesses a functional mitochondrion, which is essential for its replication. Here, we explore several fundamental aspects of the interaction between the PV and the host cell mitochondria. They include the detailed features of the congregation, the nature and mechanism of the mitochondrial travel to the PV, and the potential significance of the migration and congregation. Using a combination of biochemical assays, high-resolution imaging, and RNAi-mediated knockdown, we show that: (i) mitochondrial travel to the PV starts very early in parasite infection, as soon as the smallest PV takes shape; (ii) the travel utilizes the contractile microtubular network of the host cell; and (iii) near the end of the parasitic life cycle, when most PVs have reached their largest sustainable size and are about to lyse in order to release the progeny parasites, the associated mitochondria change their usual elongated shape to small spheres, apparently resulting from increased fission. Intriguingly, despite the well-known mitochondrial role as a major producer of cellular ATP, the parasite does not seem to use cellular mitochondrial ATP. Together, these findings may serve as foundations for future research in host–parasite interaction, particularly in the elucidation of its mechanisms, and the possible development of novel antiparasitic drug regimens.

## 1. Introduction

*Toxoplasma gondii* (*T. gondii*) belongs to the large Apicomplexan family that comprises unicellular protozoan parasites. *T. gondii* infects nearly a third of the world’s population, causing ‘toxoplasmosis’, leading to its classification by the Centers for Disease Control and Prevention (CDC), USA, as a neglected parasitic infection of major concern in public health [1]. About 40 million infections of *T. gondii* are reported in the United States alone [CDC online—https://www.cdc.gov/toxoplasmosis/about/index.html; accessed on 20 November, 2024]. In the majority of healthy and immunocompetent adults, *T. gondii* infection is essentially asymptomatic [2], whereas in others, it causes a flu-like disease characterized by myalgia, swollen lymph nodes, and fever [2,3,4]. The parasitic form that causes this self-limited acute infection in immunocompetent individuals is known as a ‘tachyzoite’ [3]. For brevity, we will often refer to the parasite as *T. gondii*, *Toxoplasma*, or Tg in abbreviation.

All intracellular pathogens manipulate the host cell for their optimal replication, which includes but is not limited to, the subversion of nutrients and the use of cellular machinery and organelles. Often, the cellular organelles are reprogrammed to serve the parasitic functions. Soon after infection, *Toxoplasma* surrounds itself with a specialized membrane-bounded cytoplasmic vacuole called the parasitophorous vacuole (PV), which performs multiple essential functions [5]. The PV membrane (PVM) acts both as a molecular sentry and molecular sieve for the parasite, allowing selective uptake of host nutrients while preventing detection of the replicating parasite by the cytoplasmic innate immune system of the host. A preliminary study using dyes of various sizes estimated that the PVM allows the exchange of molecules up to 1900 Da between the parasitophorous vacuolar space and the host cytoplasm [6].

Due to its vital role in *T. gondii* replication, we concentrated on the PVM and its interaction with one of the most prominent cytoplasmic organelles of the host cell, the mitochondria. Specifically, soon after infection, the mitochondria were found to be concentrated around the PV, which we have reproduced in our laboratory using primary human foreskin cells. From here on, we will refer to this phenomenon as PV–mitochondria association or PVMA for short. Furthermore, unless otherwise mentioned, by ‘mitochondria’, we will mean the host cell mitochondria and not the single mitochondrion of the parasite. A flurry of subsequent research over the next three decades, including some of our own, revealed various molecular aspects of PVMA and identified several cellular and parasitic factors/proteins involved in the process. Here, we focus on the molecular mechanisms of this interaction and its potential advantage to the parasite.

## 2. Results

### 2.1. Formation of Parasitophorous Vacuole and Association of Mitochondria

**Figure 1:**
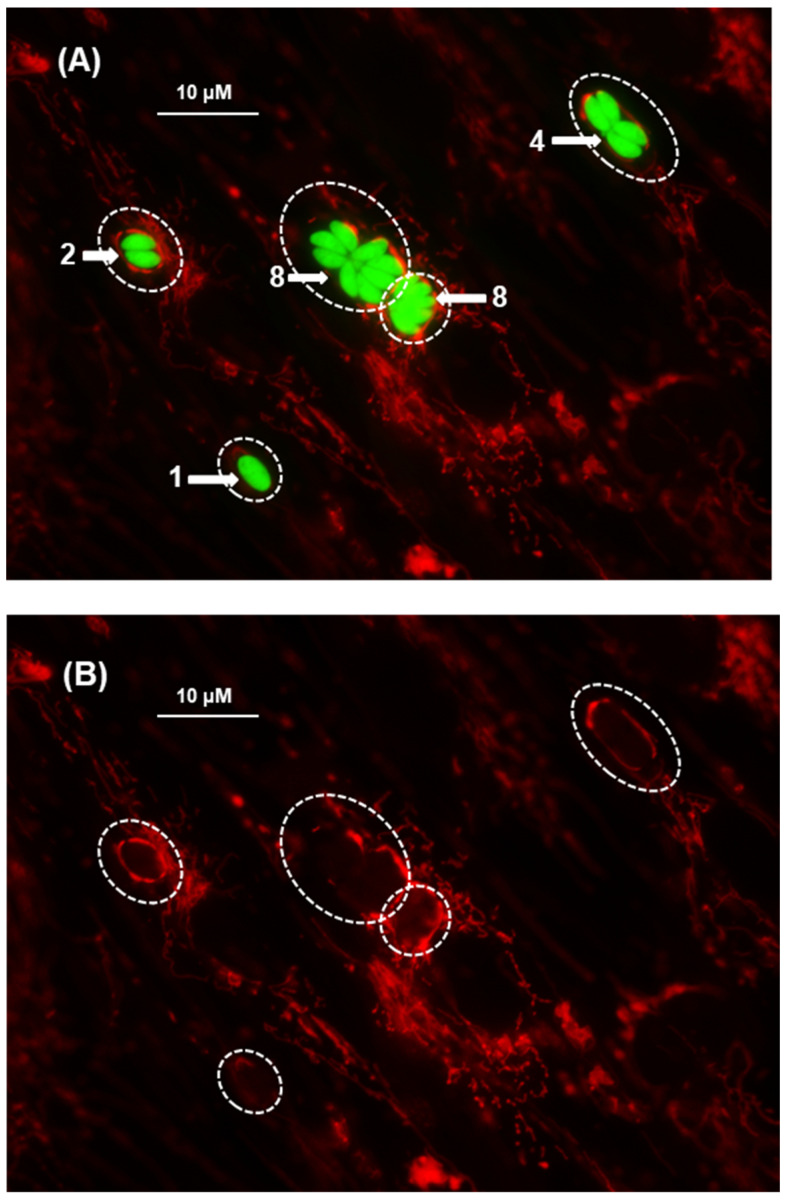
Congregation of mitochondria around the *T. gondii* parasitophorous vacuole (PV). Human foreskin fibroblasts (HFF) cells, grown in monolayer, were infected with recombinant *T. gondii* RH strain expressing Green Fluorescent Protein (GFP-Tg). Twenty-four hours after infection, the cells were stained and examined by fluorescence microscopy in a Nikon TE-2000E inverted fluorescent microscope as described in Materials and Methods. (**A**) Red = Mitochondria stained by Mitotracker Red 580; Green = GFP-expressing tachyzoites; (**B**) the same field as in (**A**), but shown without the green channel so that the PV contours can be seen more clearly. Dotted circles indicate the location of PVs. As seen, the PVs contain differing numbers of tachyzoites; the two PVs containing eight tachyzoites exhibit the characteristic rosette pattern; of these, the one on the right is tilted axially towards the viewer so that not all tachyzoites are clearly visible.

Upon entering the host cell, Tg doubles in number every 6–8 h, which continues until the host cell is fully used and destroyed [7]. *T. gondii* divides by a process called “schizogony”, in which a single initial tachyzoite gives rise to multiples of 2, i.e., 1, 2, 4, 8, 16, etc. Thus, in an asynchronized infection, different PVs contain different numbers of tachyzoites, as shown in a representative image (Figure 1).

**Figure 2:**
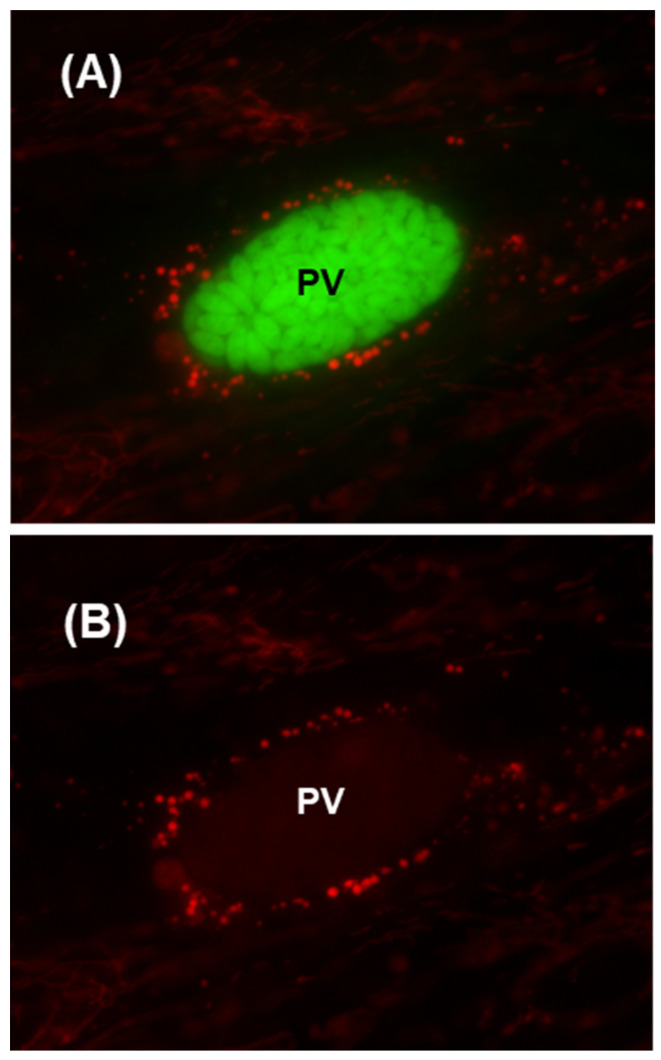
Mitochondrial alterations at the late stage of PV. About two-and-a-half days (60 h) after infection with GFP-Tg, the cells were stained with Mitotracker Red 580, and the image was captured as described in Figure 1. Note that the exact number of hours elapsed before the mitochondrial rounding is observed can vary between experiments due to small differences in variables such as the health of the inoculum, the confluency of the monolayer, and media freshness. In both (panels **A**,**B**), PV = Parasitophorous vacuole; mitochondria associated with the PV are small and rounded compared to those at earlier stages of infection (as in Figure 1). (Panel **B**) represents the same field as in (panel **A**), but without the green channel.

As the number of parasites in the PV increases, the PV also grows in size to accommodate the increasing number (Figure 1). In our experience of HFF cell infection, the highest number of tachyzoites in a PV never exceeded 128 (not shown here, but shown in Figure 2), and the PV at this point degenerates and undergoes lysis. The rupture of the cell releases the progeny tachyzoites, which then go on to infect the neighboring cells, and the process continues until all cells in the culture are completely lysed.

These results reveal multiple aspects of Tg infection relevant to PV–mitochondria interaction, namely: (i) the geometric increase in tachyzoite number along with the expansion of the PV membrane (PVM); (ii) mitochondrial congregation around the PVs; and (iii) observable mitochondrial congregation with as few as two tachyzoites in a PV, indicating that the signaling cues that promote the congregation are an early event in infection. This may also suggest a cardinal need for the association of the parasite, the host, or both. We explore it extensively in Section 2.3.

### 2.2. Change of Mitochondrial Morphology in Late Infection

Interestingly, at the late stages of replication, when the PV starts to degenerate, the associated mitochondria undergo a dramatic morphological change, becoming less extended and networked (as shown previously in Figure 1) and more rounded in shape (Figure 2). The exact reason for the switch was not pursued, but it likely indicates large-scale mitochondrial fission and mitophagy. Since this occurs at the very end of parasite replication, when the mitochondrial association may not serve any further role for the parasite, we did not pursue this phenomenon further.

### 2.3. Does the Parasite Obtain Mitochondrial ATP?

Investigations into the reasons host mitochondria congregate near the parasitophorous vacuole have been pursued from the time the phenomenon was noticed and continues to this day [8]. The PVMA has been thought to serve as a source of metabolites that the parasite draws on. ATP received our first attention in this regard since mitochondria are well known as the powerhouse of the cell, generating large amounts of ATP.

**Figure 3:**
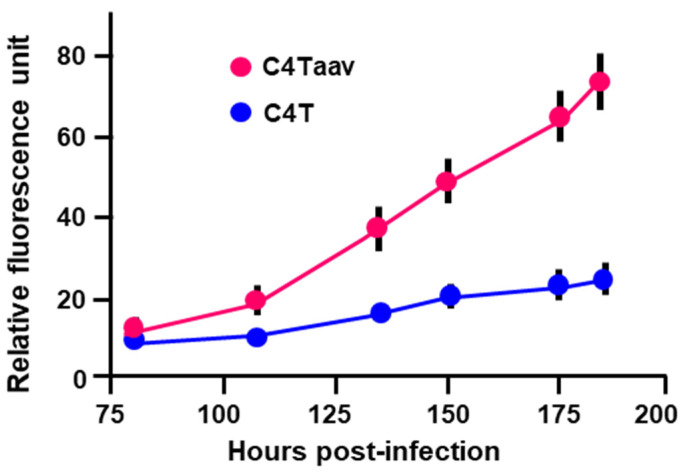
Slower growth rate of *T. gondii* in cells that are deficient in oxidative ATP synthesis. C4T or C4Taav cells were infected with GFP-Tg, and parasite replication was measured by quantification of green fluorescence as described in Materials and Methods. Relative fluorescence units at different times post-infection are plotted. C4T = Blue line; C4Taav = Red line. The results are the mean of three independent experiments, with error bars as shown.

To investigate this issue, we adopted a genetic approach in which we used a unique human osteosarcoma cell line, C4T, which carries a homoplasmic shift mutation in the mitochondrial gene encoding the ND4 subunit of NADH dehydrogenase, causing the abrogation of Complex I of the mitochondrial ETC; this prevents oxidative ATP production. As a result, C4T cells are unable to grow in media containing galactose instead of glucose [9,10,11,12]. As isogenic control, we used the C4Taav cell line, in which C4T cells were transformed with a yeast gene encoding NADH-quinone oxidoreductase, restoring respiration to two-thirds of the wild type level and allowing the cells to grow on galactose-only medium. A quantitative fluorescence growth assay of the GFP-Tg-infected cell lines showed that the overall parasite growth rate was significantly slower in the C4T cells compared to the C4Taav cells (Figure 3).

**Figure 4:**
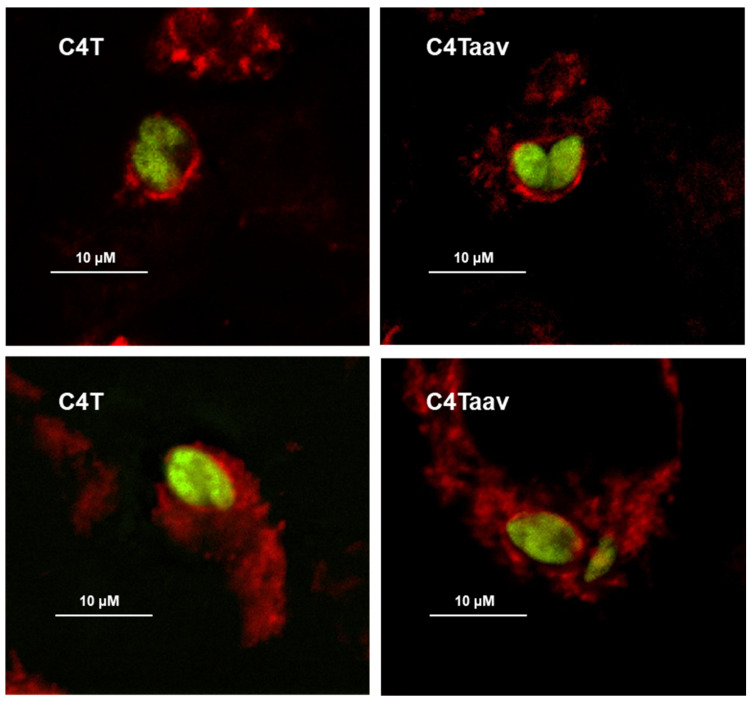
No visual difference in mitochondrial congregation with the PV between C4T or C4Taav cells. Green = GFP-Tg; Red = Mitochondria stained with Mitotracker 580. Only a few representative images are shown.

However, in visual inspection of the fluorescence images of multiple PVs, we did not see a gross difference in mitochondrial association in the vicinity of the PV between the C4T and C4Taav (Figure 4).

**Figure 5:**
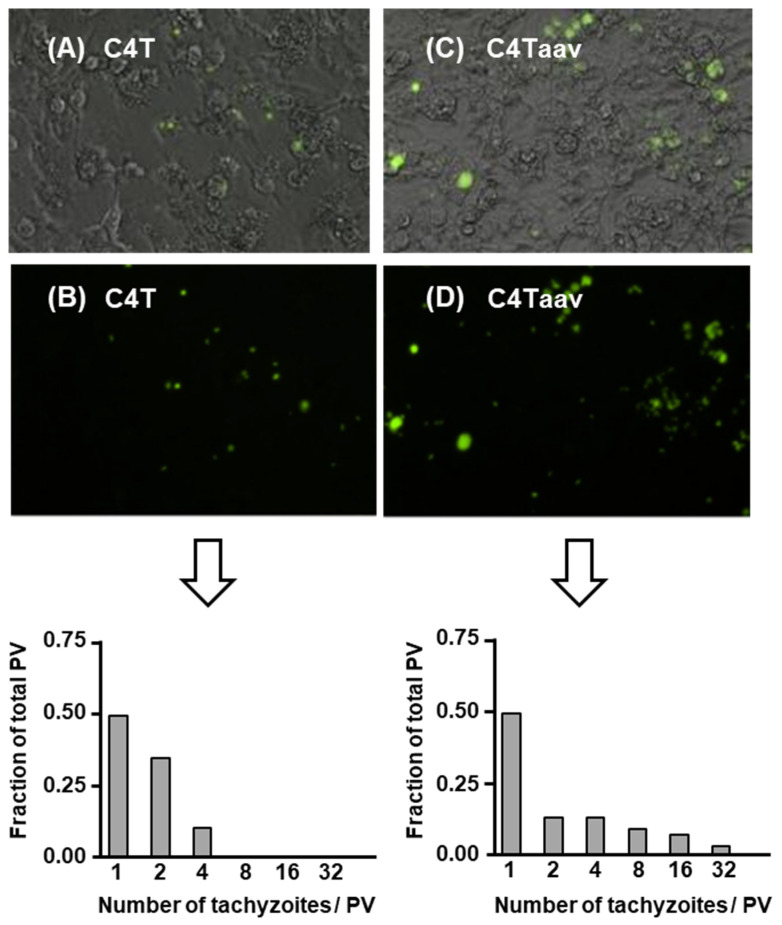
C4T (panels **A**,**B**) or C4Taav cells (panels **C**,**D**) were infected with GFP-*Toxoplasma* over a time course, and images of multiple fields were captured, two of which are presented here (Top half of this Figure). Because the tachyzoite number per PV increases two-fold every division, tachyzoite numbers can be reliably estimated based on PV size, so low-magnification images were collected to estimate the distribution of tachyzoite number per PV. Each green cluster represents a PV. (**A**,**C**) = brightfield image; (**B**,**D**) = fluorescence. A total of ~300 PVs were randomly chosen in each cell line, and the number of PVs in each size class was counted and plotted in bar graphs (Bottom half of this Figure) as a fraction of the total PVs counted.

Interestingly, whereas the infected C4Taav cells showed a normal distribution of tachyzoite number inside the PVs, ranging from 1 to 32 in the 48 h time period examined, the PVs in the C4T cells contained only one, two, or four tachyzoites (Figure 5); in other words, inhibition of host mitochondrial respiration abrogates development of the vacuoles at around the four-cell stage. However, the morphology of the stalled tachyzoites appeared to be normal.

To verify the total ATP levels in the C4T and C4Taav cells, we used a commercial luciferase-based ATP quantification assay as described earlier; to our surprise, there was no difference between the ATP content of the two cell lines. Given the abundance of cytoplasmic ATP in these cells, it appears that the PV–mitochondria association (PVMA) is not dictated by a need for the ATP derived from the aerobic respiration of the host cell’s mitochondria, at least in the C4T cell cytoplasm. We will discuss possible alternative functions of PVMA in Section 3 and Section 5.

### 2.4. Mechanisms of Host Mitochondria Localization to the PV: Role of Microtubules

A key question in the relocation of the host mitochondria to the PV is what drives the vectorial movement. We considered the role of the cellular microtubule (MT) cytoskeleton in this process since it is the principal intracellular transport machinery in the cell. To study this, we resorted to high-resolution fluorescence imaging, as detailed here.

#### 2.4.1. Optimization of Detection and Staining

First, we optimized conditions for the visualization of all three entities, namely MT, tachyzoites, and mitochondria, in Tg-infected cells. HFF cells in monolayers were infected with Tg as before, and the MTs and the mitochondria were stained with tubulin tracker (Green) and multitracker (Red), respectively. Since the tubulin tracker was green, we did not use GFP-Tg in this experiment. Instead, the Tg was visualized by differential interference contrast (DIC) brightfield, as the tachyzoites are easily recognized by their characteristic shape and rosette arrangement inside the PV (see Figure 1, for example).

**Figure 6:**
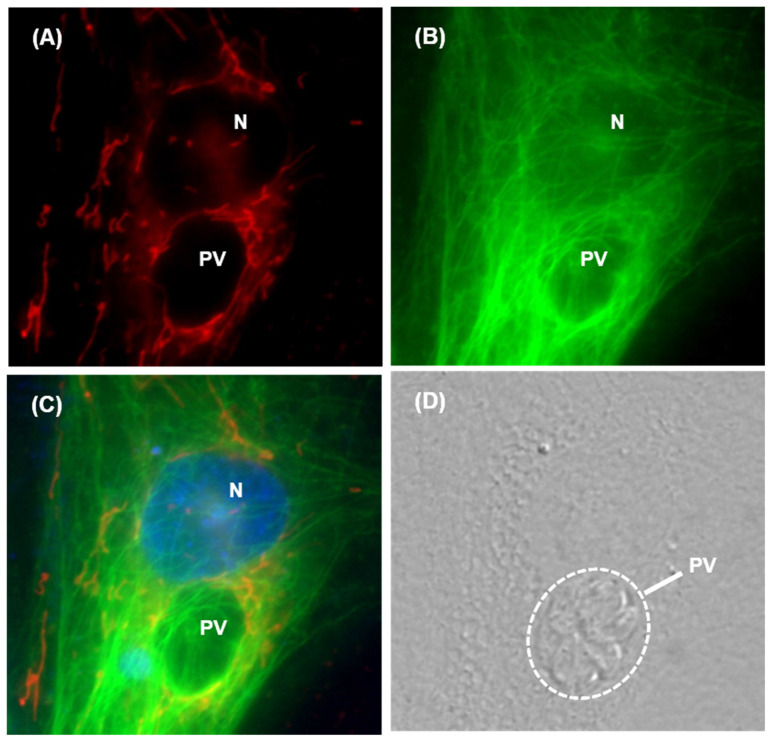
*T. gondii*-infected HFF cells at 24 h post-infection were stained with fluorescent marker dyes as described in the text and Materials and Methods and imaged live. (**A**–**C**) = Fluorescence images; (**D**) = DIC brightfield. (**A**) = Red only (Mitochondria); (**B**) = Green only (Microtubules); (**C**) = Merged image combined with nuclear staining by Hoechst 333342 (N). The parasites in this image are not fluorescently labeled but can be made out in the brightfield, in which the single PV, housing 8 tachyzoites, is circled for easy viewing.

These live imaging studies, as seen in Figure 6, demonstrate a consistently close association of microtubules and mitochondria with the PV.

#### 2.4.2. Video Microscopy of Mitochondrial Migration

**Figure 7:**
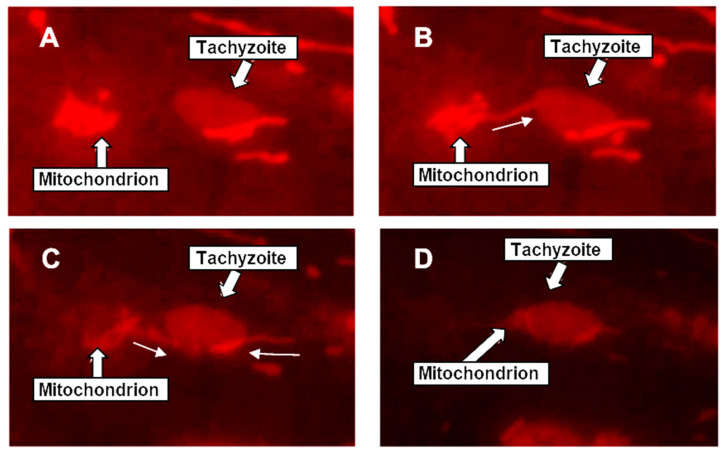
*Toxoplasma*-induced mitochondrial migration (TIMM). This set of images recorded the real-time movement of a mitochondrion to the parasite in an early-stage PV during the first hours of infection. HFF cells were infected with GFP-Tg and incubated for two hours. Cells were then stained with Mitotracker 580 and examined by live fluorescence video microscopy; frames were collected at 15 s intervals for 5 min, and representative selections are presented in the time sequence (**A**) → (**B**) → (**C**) → (**D**). Mitochondrial projections reaching towards a PV containing a single tachyzoite are indicated by the white arrowheads and followed through the time frames. Spectral leakage into the red channel from the GFP allows the tachyzoite to be visualized with the same microscope settings, facilitating rapid video microscopy. In frame (**B**), the projection from a mitochondrion attaches to the PVM and apparently remains bound. Over frame (**C**,**D**), the rest of the mitochondrion appears to be gradually reeled in and distributed along the PVM. The progressive fading of the mitochondrial stain from (**A**) to (**D**) is due to photo-bleaching from the frequent illumination needed for videography.

The preceding results suggest that the mitochondrial congregation around the PV is a nonrandom process involving host microtubules. To test that the mitochondria are indeed actively migrating to the PVM, video microscopy of infected HFF monolayers was conducted within an hour of infection, i.e., before excessive crowding would make it difficult to resolve a single event. Time-lapse video (Figure 7) showed active movement of mitochondria towards and along the PVM, a phenomenon we propose to call *Toxoplasma*-induced mitochondrial migration or TIMM.

**Figure 8:**
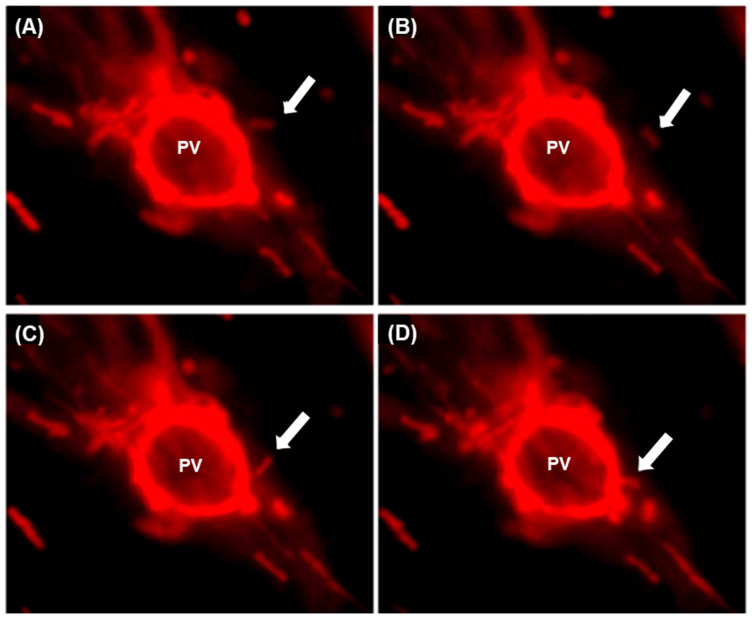
Time-lapse microscopy of infected HFF cells demonstrating a curvilinear approach of mitochondria to the PVM. Infection and imaging were performed, as in Figure 7. The frames were collected at intervals of 15 s for 5 min. In the sequence (**A**–**D**), a mitochondrion (indicated by white arrowheads) can be seen to approach and associate with the PVM. In the first image (Panel **A**). the mitochondrion is barely visible and then follows an altered path (Panel **B**), which brings it into a different plane of focus (Panel **C**). Finally, it appears to make contact with the PVM and become bound (Panel **D**).

In another video sequence of TIMM, a mitochondrion can be seen moving along a curved path towards the PVM (Figure 8A–D). MTs often exhibited a curved topology reminiscent of the curved shapes often adopted by microtubules, further suggesting that mitochondria are using MT “tracks” during TIMM. Unfortunately, longer durations of PVM–mitochondria interactions could not be readily imaged at this resolution with our instrumentation due to progressive photobleaching. In addition, there was a visible change in the morphology of the dye signal, which became more blob-like, thereby strongly suggesting that the mitochondria themselves were being damaged by the long imaging process.

In summary, these live video-microscopic studies (Figure 7 and Figure 8) revealed the kinetic aspects of TIMM, demonstrating the capability of the PV to associate with mitochondria that encounter it via mitochondrial movement. These results strongly suggest that mitochondria may be traveling along microtubule tracks to reach the PV.

#### 2.4.3. Orientation of Microtubules in Relation to Mitochondrial Migration

Each MT fiber is a bundle of protofilaments that are dimers of two subunits of the tubulin polypeptide. MTs constantly change in response to the needs of the cell by adding and subtracting tubulin dimers at both ends of the MT filament. These changes in MT length are due to the fact that one end grows faster and is called the plus end, while the other end is called the minus end. Typically, the minus end is anchored to a structure called the microtubule organizing center (MTOC), which is generally located near the nucleus. Thus, the net growth of an MT, in response to appropriate signals, occurs at the plus end. A number of cellular proteins regulate the polymerization at the plus end, one of which is EB1 (End-Binding Protein), which, therefore, acts as an indicator of MT orientation [13,14].

**Figure 9:**
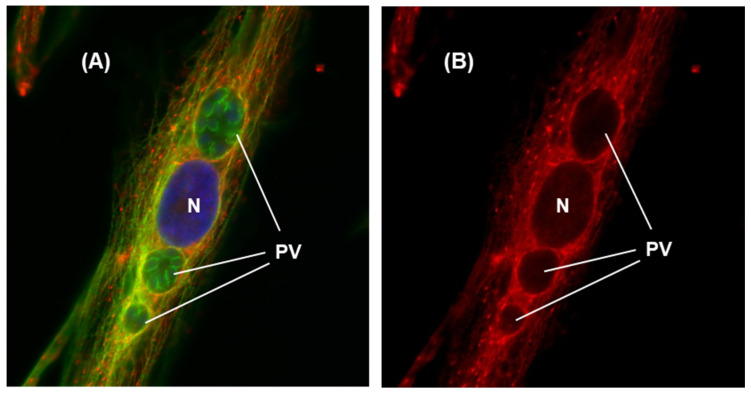
Confirmation of the specificity of the EB1 antibody. HFF cells were infected with Tg RH as described before, and 24 h later, the cells were fixed and examined by secondary (indirect) immunofluorescence microscopy. (Panel **A**) = Overlay image of nuclear (N) DNA in blue (DAPI), EB1 in red (anti-EB1 primary antibody, Alexafluor 594 secondary antibody), and microtubules in green (anti-tubulin primary antibody, Alexafluor 488 secondary antibody). (Panel **B**) = Red (EB1) only. Due to the high conservation of tubulin sequences through phylogeny, the antibody to human tubulin cross-reacts with *T. gondii* tubulin, which is visible in the parasite subpellicular and spindle regions, as evidenced by the strong green color of the parasite cells inside the PV.

*Toxoplasma gondii* itself has a highly organized microtubule cytoskeleton. To address the question of how host MTs facilitate TIMM, we needed to be able to visualize the host cell’s microtubules and not those of the parasite, so we decided to stain EB1 by indirect immunofluorescence. *T. gondii* lacks an ortholog to human EB1, so we used this as an indicator of microtubule morphology and orientation that is specific to the host cell’s microtubules. We did not observe any tachyzoite-specific staining with this antibody (Figure 9).

#### 2.4.4. Nucleation of Microtubules

**Figure 10:**
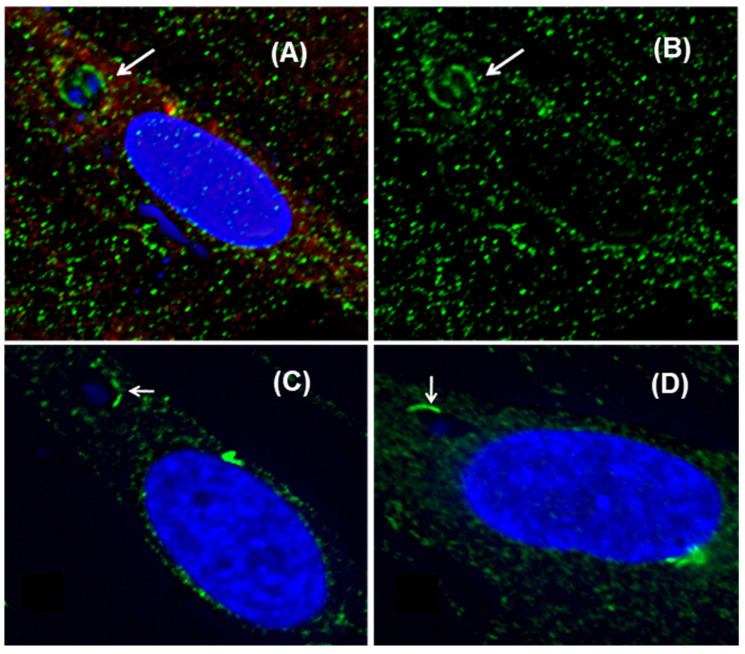
Depolymerization and recovery of host microtubules reveal nucleation of novel microtubules at or near the PVM. Human foreskin fibroblasts (HFF) were infected with RH *T. gondii*. Twenty-four hours after infection, HFF cells were placed at 4 °C for 45 min to depolymerize microtubules and then placed at room temperature and fixed in methanol at intervals. (Panels **A**,**B**) show an infected cell fixed immediately after cold treatment, demonstrating depolymerization of microtubules stained with anti-tubulin (green) and anti-EB1 (red) antibodies, and DNA stained by DAPI (blue). (Panel **A**) shows a merged image of all three channels, while (**B**) shows tubulin alone. The arrow indicates cold-resistant microtubule staining in Tg tachyzoites. To focus exclusively on host microtubules, cells in (panels **C**,**D**) were assayed by immunofluorescence microscopy against human EB1 (green), and co-stained with DAPI (blue). (Panels **C**,**D**) are representative images of infected cells at 4 min post-release from 4 °C, and the arrows indicate nascent human microtubules forming at or near the PVM. The positive staining located near the host nucleus represents the outgrowth of EB1-bound microtubules from the microtubule organizing center (MTOC).

Given the association of host microtubules with the PVM and the structural directionality of microtubules, we inquired whether *T. gondii* infection induced the nucleation of new host microtubules at the PVM, which could benefit the parasite by ensuring a predictable orientation of some captured host microtubules. To visualize only newly nucleated microtubules at the PVM, we infected HFF cells as before and, at 24 h post-infection, performed a cold-induced microtubule depolymerization and recovery assay. In this procedure, the cells are first exposed to 4 °C for 45 min, a condition that depolymerizes microtubules, and then allowed to recover at room temperature and fixed at intervals. Normally, host microtubules are polymerized from the MTOC outwards. If *T. gondii* nucleates new microtubules, then the latter should be visible at the PVM following recovery from 4 °C. The results (Figure 10) showed that this is indeed the case; nucleation of new microtubules occurred within a few minutes of release from the depolymerized state. Newly forming microtubules from the host MTOC are also visible as brightly stained areas near the host nucleus.

**Figure 11:**
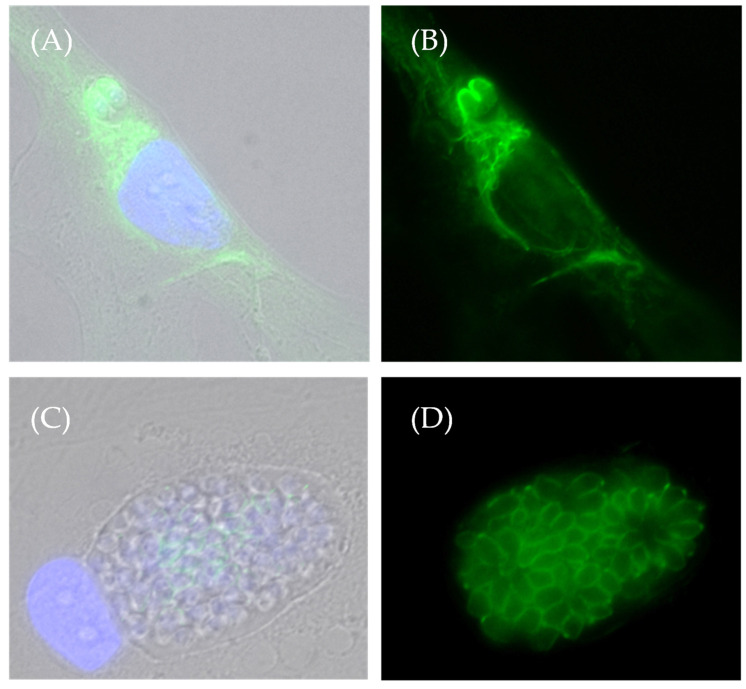
Human foreskin fibroblasts (HFF) were infected with the *T. gondii* RH strain and then fixed and examined by immunofluorescence microscopy against acetylated tubulin (anti-acetylated α-tubulin primary antibody and Alexafluor 488 secondary antibody). Both the early-stage (panels **A**,**B**) and the late-stage (**C**,**D**) infections show a prominent staining of the tachyzoites for acetylated tubulin. Panels on the left show a merged overlay of DAPI (blue), acetylated tubulin (green) and DIC brightfield images; panels on the right show green fluorescence alone. Interestingly, in early infection (**A**,**B**), a subpopulation of HFF microtubules is acetylated, but by late infection (**C**,**D**), the host microtubule cytoskeleton is no longer intact, and no staining of host microtubules is observed.

During these experiments, it was also noted that while the host microtubules were completely depolymerized by cold, the anti-tubulin antibody still stained the *T. gondii* microtubules, readily seen in the pellicular regions (Figure 10). This was also observed during nocodazole-induced depolymerization of microtubules. This suggests that Tg microtubules might be resistant to perturbations that depolymerize microtubules, perhaps due to post-translational modifications. Acetylation of microtubules, in particular, has been reported to convey resistance to cold-induced depolymerization [15], and post-translational modifications of microtubules are highly regulated in tissue- and species-specific manners [16,17]. Therefore, we stained infected HFF cells with an antibody specific for acetylated tubulin and observed that the pellicular regions of the tachyzoites were prominently stained by this antibody (Figure 11). This correlates with the location of the tubulin-positive stain in depolymerizing conditions, as seen in Figure 10A,B).

#### 2.4.5. Assay of PV-Microtubule Association: Effect of Nocodazole

**Figure 12:**
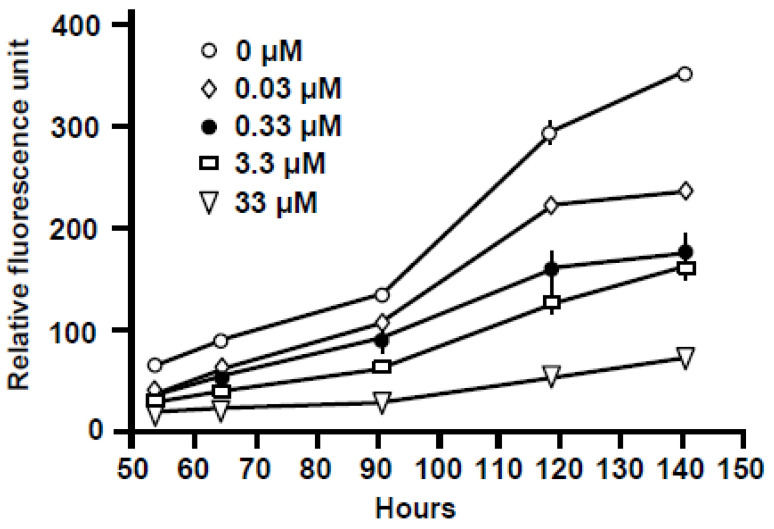
Dose-dependent inhibition of Tg growth by the microtubule depolymerizing agent, nocodazole. HFF cells were grown in 96-well plates, treated with the indicated concentrations of nocodazole for 2 h, and then infected with GFP-Tg. Total GFP fluorescence was measured for each well at intervals of 12 h, as described under Materials and Methods (Section 6.3), and plotted in relative fluorescence units as shown. The standard error bars are derived from three experiments; where the error is very small, the bars are not visible due to being smaller than the symbols indicating the dose.

To test if PV–microtubule association is important for the replication of the parasite, we investigated whether depolymerization of microtubules prevented PVMA and parasite growth. Nocodazole is a well-known chemical that induces MT depolymerization and/or suppresses MT dynamics in various cells [18]. As shown (Figure 12), nocodazole inhibited Tg growth in a dose-dependent manner. Interestingly, growth was not completely inhibited, even at 33 μM, when cytotoxic effects on the host cells were observed.

**Figure 13:**
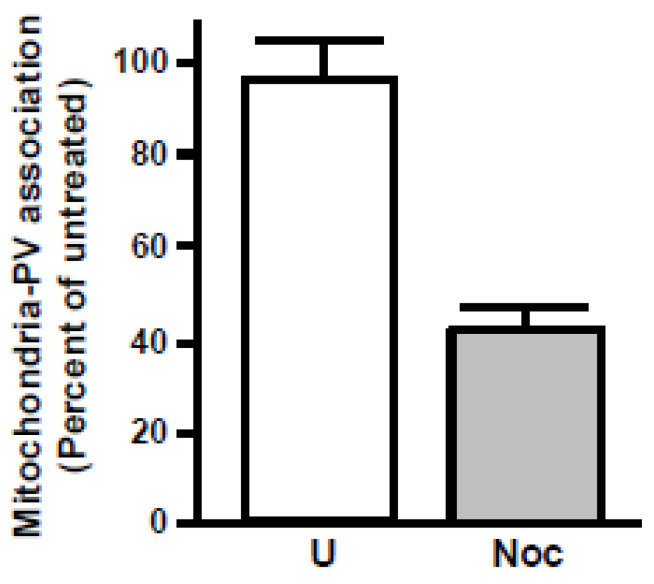
Inhibition of PV–mitochondria association (PVMA) by the microtubule depolymerizing agent, nocodazole. HFF cells were grown in 96-well plates, treated with the indicated concentrations of nocodazole for 2 h, and then infected with GFP-Tg. Twenty-four hours after infection, the cells were stained with Mitotracker 580, and a quantitative assay of PVMA was performed as described (Section 6.5). The numbers were plotted as a percentage of the PVMA in untreated (U) cells. We performed the assay with a range of nocodazole concentrations, but for the sake of brevity, only the result with the highest concentration of the drug used in Figure 12 (33 μM) is shown. The standard error bars are derived from three experiments.

To see if the growth inhibition effect of nocodazole is reflected in reduced mitochondrial accumulation around the PV, we developed a custom, quantitative algorithm based on “Regions of Interest” (ROIs) as described in Section 6.5 with an illustration. The final results clearly showed a statistical reduction of PVMA in the drug-treated cells compared with untreated controls (Figure 13).

Based on these results, it appears that the reduction of parasite growth by nocodazole correlates with the reduction of PV–mitochondria association.

#### 2.4.6. Inhibition of *T. gondii* Growth by Knockdown of Microtubule Motor Proteins

Dynactin is a multiprotein complex necessary for the function of the microtubule motor protein dynein [19,20,21]; DCTN2 (dynactin subunit 2; also known as dynamitin) is an essential subunit of the complex [22]. Dynein is responsible for the minus-end movement along microtubules, and as a subunit of this complex, DCTN2 is involved in anchoring microtubules to cognate structures, such as centrosomes [23]. In the presence of other accessory factors, DCTN2 activates the complex to move along the microtubules over long distances [24,25].

**Figure 14:**
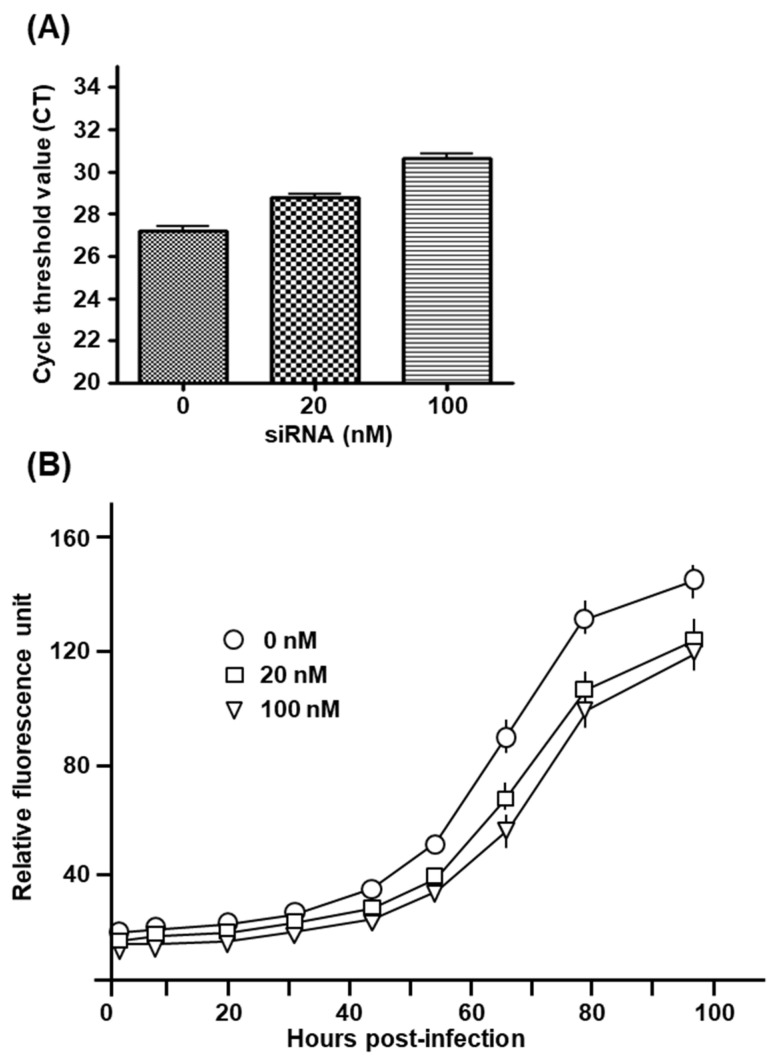
Loss of DCTN2 (dynamitin) inhibits *T. gondii* growth. siRNA transfection is described in Materials and Methods. We used two concentrations of siRNA as shown, viz. 20 nM and 100 nM siRNA. In the ‘no-siRNA’ control (0 nM siRNA), sterile buffer replaced siRNA in the transfection mix. (**A**) At 36 h after transfection, the total RNA of the cells was subjected to a quantitative reverse transcription-polymerase chain reaction (qRT-PCR) using the SYBR Green-based assay from Bio-Rad (as described in Section 6), and the CT values, normalized against GAPDH CT, were plotted. Each bar represents the mean of three CT measurements, with the standard error as shown. The CT values of the three measurements (as plotted here) were: 0 nM = 27.5, 27.6, 26.8; 10 nM = 28.8, 28.4, 29.1; 100 nM = 30.3, 31.1, 30.5. (**B**) In parallel wells, the transfected cells were infected with GFP-Tg; total fluorescence was measured for each well at 8–20 h intervals, and relative fluorescence values were plotted. Standard error bars from triplicate experiments are also shown; where the error is very small, the bars are not displayed in order to avoid cluttering.

We reasoned that if the MT motor function is required for Tg-induced mitochondrial movement (TIMM), PVMA, and Tg growth, then the corollary is that inhibition of the MT motor function may also inhibit Tg growth. To investigate this, we knocked down DCTN2 by RNA interference, using a pool of four validated siRNA targeting DCTN2 mRNA, as described in Materials and Methods (Section 6.6). We confirmed that DCTN2 protein levels were, in fact, reduced by the siRNA pool (Figure 14), which resulted in a modest but appreciable inhibition of overall parasite growth.

**Figure 15:**
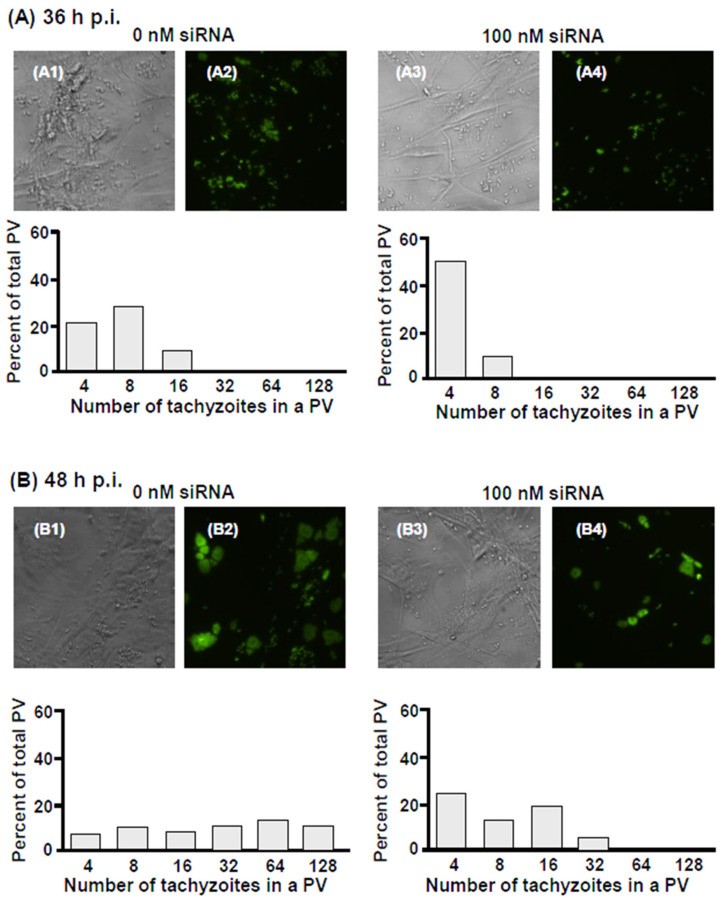

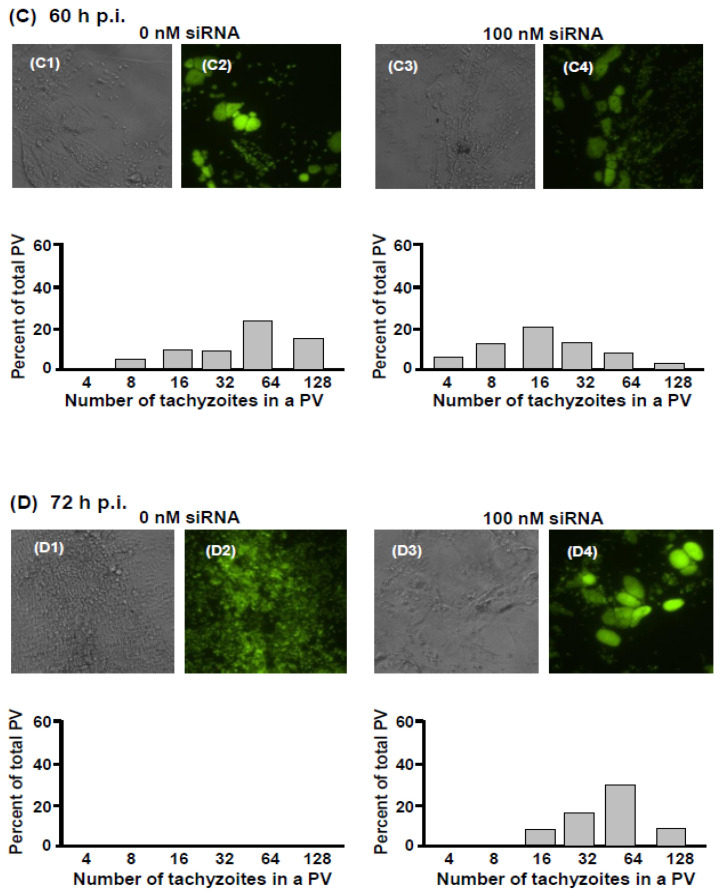
HFF cells were transfected with 0 or 100 nM of DCTN2 siRNA and 36 h later infected with GFP-Tg, as described in Figure 13. The cells were followed with time, and images were captured at the indicated times (36 h, 48 h, 60 h, and 72 h post-infection), respectively, in (panels **A**–**D**). In each pair (e.g., **A1**,**A2** pair, or **A3**,**A4** pair), the phase-contrast image is on the left and the fluorescence image, is on the right. PVs were binned for the number of tachyzoites they contain (four, six, eight etc., as shown), and their numbers were expressed as percent of total PV counted, as also described in Figure 5.

Recall that in our studies comparing Tg growth between C4T and C4Taav cells, we discovered a profound difference in the number of tachyzoites that the PVs in the two cell lines could reach, which indicated a kinetic block in parasite cell division in host cells deficient in aerobic respiration. We asked if such a division defect also occurs in DCTN2 deficiency. The experiment was performed in a similar fashion, whereby we infected the DCTN2 siRNA-treated HFF cells with GFP-Tg and counted the number of tachyzoites in each individual PV over 72 h of infection. ‘No-siRNA’ cells were used as controls. Based on the optimization of DCTN2 knockdown (Figure 14A), we used only 100 nM siRNA, and the results are presented here (Figure 15A–D).

These results (Figure 15A–D) revealed that at any time post-infection, the DCTN2 knocked-down cells have a lower number of tachyzoites in their PVs (compared to the control cells), suggesting that the kinetics of parasite division were negatively affected by the loss of DCTN2. For instance, at 36 h p.i. (Panel A), the siRNA-untreated cells included 16-cell PVs, while the DCTN2 knocked-down cells did not have any observable PVs with more than eight cells. This is also consistent with the greater overall fluorescence intensity (for GFP-Tg) in the untreated cells compared to treated cells at the same time point. By 72 h p.i. in untreated cells (Panel D), virtually all PVs had lysed, and the monolayer was disrupted, leaving no intact PVs to count. We conclude that the dynein complex, in general, and DCTN2, in particular, are required for TIMM and for optimal *T. gondii* growth and replication.

## 3. Summary

The principal finding of this paper is the important role of the host cell ‘s microtubule cytoskeleton and mitochondria in the growth and replication of *Toxoplasma gondii*, a clinically significant parasite. We have used two complementary approaches to demonstrate this role, namely nocodazole treatment and RNA interference, during which we also showed that mitochondrial association with the PV membrane (PVM) is dependent on the host MT and a functional dynein/dynactin complex. Together, these findings also suggest that *T. gondii* does not merely passively associate with mitochondria as a result of its growth or binding of host MT, but in fact, it occurs through TIMM that entails mitochondrial travel towards the PV via minus-end-directed microtubule motors. We also presented evidence that microtubules are nucleated at the PVM, potentially causing minus-ended travel along these MTs to converge at the PVM. We conclude that TIMM is important or even essential for the completion of the *T. gondii* life cycle.

## 4. Limitations of This Study

While the results of this study are summarized above (Section 3), we can perceive a few limitations, as described here briefly (Section 5).

(a) Ideally, it would be desirable to have a dynamic view of the multipartite interaction between mitochondria, microtubules, and the PVM. This would require the use of a multi-laser confocal microscope with finer resolution of the emission spectra and long-term video-microscopic capability that would also prevent bleaching of the fluorophore. This was not possible at the time of our study but is certainly feasible in the future.
(b) In the same vein, we were far from identifying all the interacting partners of the incoming mitochondria and the PVM, which will also be required to fully understand the molecular events pertaining to PVMA.
(c) Even though several laboratories have shown that *Toxoplasma* scavenges host mitochondrial lipids, such as cardiolipin, it still remains an enigma as to why ATP was apparently spared, particularly since the rapid replication of the tachyzoites must consume large amounts of energy, which is best obtained through aerobic respiration.
(d) Due to the limited scope of this study, we left out the mechanism of mitochondrial change of shape when the tachyzoites are nearing egress. We speculated but could not address if the process is associated with mitophagy.

## 5. Discussion

Our findings are in agreement with several earlier reports in the literature, and they also complement them. Several studies, including one in the 1980s, microscopically observed cultured cells infected with *T. gondii* and noted changes in the distribution of the cellular mitochondria [26,27,28,29]. A classic study [30] that used mouse macrophage cells and specific organelle markers indicated that the PV–mitochondria association (PVMA) requires active invasion and not internalization by general phagocytosis since the PVM lacks indicatory host membrane proteins such as phagocytic receptors. However, once the association had occurred, killing the parasite by treating the infected cells with pyrimethamine, an established microbicide, had no effect on the association. Thus, parasite activity is needed for the establishment but not for the maintenance of PVMA. These studies revealed factors not responsible for association but did not identify those that were; we have shown that the MTs play this role at least in part. Various other studies implicated *Toxoplasma* factors important for PVMA, the most notable of which is MAF1b (Mitochondrial Association Factor 1b), discovered by a combination of genetic and biochemical approaches [29,30,31].

Regarding the need for mitochondrial recruitment to the PVMA and their stable association, we have only explored whether this is an avenue for the parasite to receive host cell ATP (Section 2.3). We observed a kinetic difference in *Toxoplasma* growth between the C4T and C4Taav cells (Figure 3), but there was no difference in the ATP content of these cells. However, we did not actually measure the ATP contribution from the mitochondria; if this contribution were only a small fraction of the total cellular ATP, it might not have been appreciable in our quantification, but its loss might still affect the kinetics of parasite growth. In fact, the effect seemed very strong, in the sense that no PV contained more than four tachyzoites (Figure 5), even though we imaged infected cells for extended periods, up to 195 h post-infection, at which point the monolayers lost integrity, producing cell debris and making imaging impossible. To integrate these findings, we conclude that the parasite’s apparent requirement for host mitochondria is not exclusively based on ATP produced by oxidative respiration within those mitochondria.

Other researchers have identified the use of host mitochondrial lipids by the parasite. *Toxoplasma* replicates almost continuously within the host cell and, therefore, needs lipids for the biogenesis of membranes, including the PVM. Very early studies, in fact, suggested that the bulk of PVM lipids are derived from the host [32]. Subsequent studies revealed that the scavenged lipids comprise a large variety, such as long-chain fatty acids, cholesterol, and phospholipids such as phosphatidylcholine and cardiolipin [33,34,35,36,37,38,39]. The details of lipid transfer from the host to the PVM remain to be elucidated.

The PVM-associated host mitochondria gradually change morphology during infection, adopting a rounded appearance (Figure 2). Mitochondria constantly change their length by fusion and fission, regulated by specific proteins. It has been recently shown that the association of mitochondria with MTs inhibits mitochondrial fission by precluding the assembly of the fission protein Dnm1 [40]. When the cells are nearing lysis for parasite release, the MT filaments are also largely dismantled and presumably are no longer needed to hold the mitochondria at the PVM. This should allow Dnm1 engagement, promoting mitochondrial fission, which may explain the conversion of the mitochondria to a small, round shape. The eventual fate of these mitochondria remains an interesting but unexplored subject.

Lipoic acid (LA), also known as α-lipoic acid, is another lipid that the parasite obtains from host mitochondria, and here, the MT-mediated transport of the mitochondria to the PVM may facilitate the uptake. LA is a sulfur-containing fatty acid with antioxidant properties and is involved in an extremely rare post-translational modification (known as lipoylation), which, in mammals, has been found in only four multimeric metabolic enzymes [41,42]. Nevertheless, lipoylation is considered profoundly important since these enzymes and their lipoylation sites are conserved throughout the evolutionary kingdoms. A collaborative study in 2006 [43] demonstrated that in *T. gondii*, the apicoplast is the only site of LA synthesis, which is consistent with the ability of plant chloroplasts to synthesize lipoic acid [44] and the chloroplast origin of the apicoplasts [45]. Although the biosynthesis of lipoic acid in eukaryotes occurs in the mitochondria, the *Toxoplasma* mitochondrion has obviously lost this ability, perhaps due to the acquisition of the apicoplasts.

However, when the apicoplast LA synthesis was disrupted by a mutation in the biosynthetic pathway, lipoylation in the parasite mitochondrion continued unaffected. When the *T. gondii*-infected cells were grown in an LA-deficient medium, lipoylation of mitochondrial (but not apicoplast) proteins was strongly reduced, which was rescued by the addition of LA to the medium. The simplest conclusion from these results [43] is that the LA synthesized by the apicoplast is insignificant compared to the much larger needs of the parasite mitochondrion, which are fulfilled by the scavenging of the abundant supply of LA in the host mitochondria.

Yet another leading study [46] noted a different role of MTs for the PV, in which the host endocytic structures were translocated along the MTs to the PV and were finally delivered into the vacuolar space by MT-based invagination of the PVM. When the MT function was inhibited by treatment with taxol, an MT-depolymerizing agent, the endosomes were never delivered inside the PV. It was proposed that this function of the MTs allows parasite access to diverse components from host digestive compartments.

In our work, we have focused on the role of the microtubules, but at least two studies pointed to the role of the cellular intermediate filaments in *T. gondii* growth, particularly PVM–host interaction [46,47]. Together, these two studies showed that vimentin, a constituent of intermediate filament (IF), undergoes changes upon infection [46] and accompanies the MTs in the infected cell [46]. Thus, the TIMM that we have demonstrated here appears to help transport or deliver several host metabolites.

In spite of major progress in our understanding of PV–host interactions, it seems that we have only seen the tip of this iceberg and that much more remains undiscovered. In the PVMA studies, the amino acids or domains of the interacting pairs of PVM surface and tubulin have not been identified, thus limiting our knowledge of the molecular aspects of the interaction. The large amount of work that is yet to be done certainly portends an exciting time of discovery in this area. We believe our results will add to the foundation for future studies.

In terms of clinical contribution, molecular characterization of the mechanism of TIMM might lead to a new therapeutic modality for *Toxoplasma* infection. It may also throw new light on diseases caused by reduced MT stability and dynamics, which are generally also accompanied by abnormal mitochondrial location; examples include several neurodegenerative diseases such as Alzheimer’s disease, Parkinson’s disease, and Amyotrophic Lateral Sclerosis (ALS) [48,49].

## 6. Materials and Methods

### 6.1. Cells

Primary human foreskin fibroblast (HFF) cells were isolated using the laboratory protocol of Dr. Boris Striepen (University of Pennsylvania, Philadelphia, PA, USA), and were used in all our experiments. In brief, foreskin tissue was obtained following the circumcision of anonymous infant donors. The tissue was first washed in phosphate-buffer saline (PBS) and then diced using sterile scissors. The diced tissue was incubated with 5% trypsin solution for 2 h and then diced again. Fresh MEM (Minimum Essential Medium) containing 10% fetal bovine serum (FBS) and 10% penicillin-streptomycin was added to the tissue; the mixture was placed in a 75 cm^2^ culture flask and incubated at 37 °C and 5% CO_2_ until cells morphologically resembling fibroblasts were observed growing out from the tissue chunks. When the cells had reached 100% confluency, the cultures were incubated with trypsin and passed onto new flasks, avoiding as much tissue chunk as possible. Growth and passage were repeated until a uniform monolayer of cells was obtained. These cells were used in experiments, and frozen stocks were also prepared by resuspending the cell pellets in a growth medium containing 10% dimethylsulfoxide (DMSO) and then stored in cryotubes at −80 °C in small portions so that the number of future passages was kept at a minimum. All cell culture reagents were from Gibco (Grand Island, New York, NY, USA)

### 6.2. Parasite

The virulent *Toxoplasma* RH strain (Type I) was used in all experiments. The parasite was grown on HFF cells, essentially as described [50], with minor modifications. Following complete lysis of the monolayer, the egressed tachyzoites were purified by filtration through polycarbonate filters of 3–4 μm pore size (Nalgene Nunc International, Rochester, NY, USA) to separate them from host cell debris and further purified by differential centrifugation at 5000× *g*. The parasite pellet was resuspended in an ice-cold infection medium containing 10% DMSO and stored frozen at −80 °C in small portions [50].

### 6.3. Fluorescence-Based Parasite Growth Assay

HFF cells were grown in monolayers in a 96-well, black-walled, clear-bottomed plate. Experiments and infections with GFP-Tg were performed in these plates, and fluorescence was measured in a SpectraMax M5 plate reader (Molecular Devices, LLC., San Jose, CA, USA), with the following settings: Excitation, 480 nm; Emission, 540 nm; filter cut-off, 530 nm. These settings yield a background fluorescence of ~25 units in uninfected HFF cells, which was subtracted from the experimental readings. Fluorescence was collected every 12 h for infected monolayers, and the resulting growth curves were compared using nonlinear regression analysis.

Note that while the HFF monolayers remain intact for many days, even a week or longer, the Tg-infected monolayer starts to completely degrade around 4–6 days, or when the highest number of tachyzoites in some PVs has reached the 32–128 range. However, the exact time of lysis may differ somewhat in different experiments, perhaps depending on factors such as the passage number of the cells, the age of the FBS, and the storage history of the parasite.

### 6.4. Microscopy

Images were captured in the Nikon TE-2000E inverted microscope (Melville, NY, USA) located in the Lions/University of South Alabama Eye Research Center in the Frederick P. Whiddon College of Medicine. The microscope is equipped with 10×, 20×, 40×, and 60× objectives with the capacity for phase contrast and differential interference contrast (DIC) for some objectives. The objectives were chosen as needed for the experiment, and focusing was performed either manually or using Nikon’s Perfect Focus system. For samples with greater depth of field, multiple focal plane images were merged by Z-stacking.

Samples were excited with the EXPO X-cite Series 120 fluorescent lamp, which was part of the microscope, and emitted light was filtered with a standard filter set for detecting DAPI/FITC/TRITC and other fluorophores with comparable emission spectra. For live cell imaging, a stage-top incubated culture chamber with controlled temperature and CO_2_ setting was used, which allowed for time-lapse video microscopy. All time-lapse images were taken with a 60× oil-immersion objective and the Perfect Focus system, which utilizes an IR light source to detect the media–air boundary in the sample and maintain a constant Z-depth relative to this boundary. All images were captured with a Roper CoolSnap camera (Tucson, AZ, USA) connected to a PC running the NIS Elements software package (v. 5.21.00), which was also used for image processing and analysis. The NIS elements package is a proprietary software of Nikon, and was provided along with the microscope mentioned above. Custom measurement algorithms were programmed using the macro-scripting language of NIS Elements.

Live cell imaging was conducted either in the growth/infection media or in KGD buffer, which contained 0.18 g d-glucose and 5 g of dextran in Krebs buffer (118 nM NaCl, 25 mM NaHCO_3_, 4 mM KCl, 1.2 mM KH_2_PO_4_, 1.3 mM CaCl_2_, 1.2 mM MgSO_4_, pH 7.4).

Mitochondrial imaging was performed by incubating monolayers for 35 min with 150 nM Mitotracker in the growth or infection medium. Monolayers were then washed with PBS, pH 7.4, and the growth/infection medium was replaced. Cells were then imaged with the fluorescence lamp set to 12% power to prevent photobleaching.

Microtubule imaging was performed by incubating monolayers with 150 nM of Tubulin-Tracker in KGD buffer, after which the monolayers were washed once and then viewed.

Nuclei (DNA) imaging was performed by incubating monolayers with 500 nM of Hoechst 33342 dye (R&D; Systems, Minneapolis, MN, USA) in either growth medium or KGD buffer for 35 min at 37 °C and 5% CO_2_. Following incubation, monolayers were washed and viewed in the appropriate buffer. All three of the above stains were combined in a single step using KGD buffer as the staining and viewing buffer where appropriate.

For immunofluorescence studies, cell cultures were grown in covered, glass-bottom dishes, and experiments were performed in these dishes. Cells were then washed with PBS and placed in 100% ice-cold methanol at −20 °C for 4–6 min. Methanol was removed, and monolayers were rinsed with 0.1% Triton X-100 in PBS. Cells were then incubated in 10% normal goat serum (NGS) in PBS for 45 min. PBS + NGS was then removed, and cells were incubated with primary antibodies for 2 h. Cells were then washed with PBS five times and incubated in the presence of secondary, fluorophore-complexed antibodies for 45 min. Cells were then washed again five times in PBS, and a coverslip was then mounted onto the glass dish using the VECTASHIELD mounting medium, either with or without DAPI (Vector Labs, Newark, CA, USA). Mounted dishes were incubated in the dark at room temperature overnight and visualized the next day with the Nikon TE-2000E inverted fluorescent microscope, equipped with standard filter sets. Microtubules were visualized by secondary immunofluorescence using polyclonal rabbit antibodies raised against alpha- and beta-tubulin (Sigma-Aldrich, St. Louis, MO, USA). The plus-end-associated protein EB1 was visualized using a rabbit polyclonal antibody raised against human EB1 (Santa Cruz Biotechnology, Dallas, TX, USA). Images were captured as described earlier in this section.

### 6.5. Image Processing for PVMA Assay

**Figure 16:**
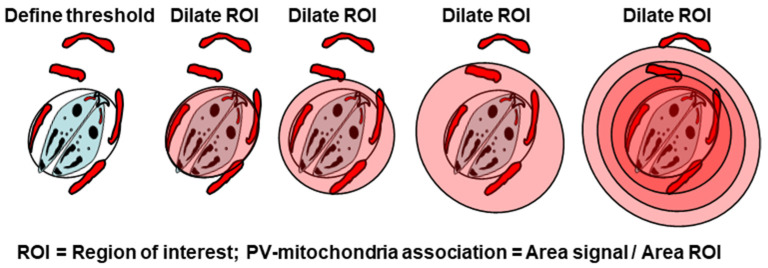
Schematic representation of our workflow for quantifying relative PVMA.

PV–mitochondria association (PVMA) was quantified using a custom algorithm programmed as a macro in NIS Elements, and the workflow is depicted here (Figure 16). This algorithm worked by measuring the area around the PVM and defining PVMA as the percentage of this area occupied by mitochondrial signal. First, an image was selected for analysis, and a threshold was determined and applied to the image to define the Mitotracker signal. This yielded a binary layer whose attributes were measured. To define the PVM, a region of interest was drawn along the PVM. This region of interest was then dilated, preserving the shape of the PVM as defined in the step above. This operation was repeated twice to generate three concentric regions around the PVM. At each step, the fraction of the area of the ROI that was occupied by the mitochondrial signal was measured, as defined by the formula: Association = Area signal/Area ROI. These values were then compared between experimental conditions to assay changes in PVMA. This and all other experiments were performed in triplicate and subjected to statistical analysis using either a student’s *t*-test for experiments with two conditions or a one-way analysis of variance (ANOVA) for experiments with more than two conditions.

### 6.6. Knockdown of DCTN2 (Dynamitin) by siRNA

To knock down the expression of DCTN2 by RNA interference, HFF cells were transfected with “ON-TARGETplus SMARTpool” siRNA (Short Interfering RNA) from Dharmacon, as described below.

The four siRNAs targeted the following sequences in DCTN2 mRNA (GI: 1124890756) (all written 5′ to 3′):

UUAUGAAACUAGCGACCUA,
UCUCAGAUCGUAUUGGAAA,
GGACAAUACCACCCUCUUG,
GAGGACAGGAUAUGAAUCU

The siRNA pool was transfected with SiPort Amine transfection reagent (Ambion, Austin, TX, USA). Infection of siRNA-treated HFF monolayers was performed at 36 h post-transfection. For transfection, we used the “reverse” transfection method, in which the RNA-reagent complexes were added to a culture plate, and cells in suspension were plated into the mixture [51].

The siRNA treatments were performed either in glass-bottom dishes (for PVMA assay) or in clear-bottomed black 96-well dishes (for growth assays). A newly-confluent monolayer of HFF was trypsinized and resuspended in a normal growth medium and then incubated at 37 °C. The transfection complexes were prepared as follows. First, the transfection reagent was allowed to come to room temperature and was then added to OPTI-MEM minimal essential medium and incubated at room temperature for 5–10 min. The siRNA was then resuspended in OPTI-MEM and added to the transfection reagent complex and incubated for 35 min, as suggested by the manufacturer (Ambion). Complexes were then placed in the center of the growth surface, and the HFF cells in suspension were pipette-mixed and plated on top of the siRNA/reagent complexes at a confluency of 50%. Culture dishes were placed at 37 °C. At this point, total RNA was collected 24 h post-transfection for qRT-PCR analysis (described below) using the RNeasy kit (Qiagen, Germantown, MD, USA).

For the qRT-PCR assay of knockdown, total RNA was isolated, and the concentration of RNA was assayed with a one-drop spectrophotometer. qRT-PCR was performed with an iCycler (Bio-Rad Laboratories, Hercules, CA, USA) Thermocycler/Luminometer using the SYBR-Green RT-PCR kit (Applied Biosystems, Foster City, CA, USA) according to the manufacturer’s instructions. The CT value for the housekeeping gene GAPDH was used as a control, and calculations were done using the manufacturer’s software. The primers for qRT-PCR were as follows.

DCTN2:

Forward 5′-ACTTGGATACCACCCAGCAG-3′,

Reverse 5′-AACTGTGGCCAGGTTTTCAC-3′;

GAPDH (Glyceraldehyde 3-phosphate dehydrogenase):

Forward 5′-CCAAAAGGGTCATCATCTCTGG-3′,

Reverse 5′-ATTTGGCAGGTTTTTCTAGACGG-3′.

### 6.7. Microtubule Depolymerization and Recovery

To determine if *T. gondii* nucleates novel host microtubules, monolayers of HFF cells grown in glass-bottomed dishes were first exposed to conditions that depolymerize microtubules (i.e., either subjected to temperatures below 4 °C or treated with nocodazole), and then allowed to recover from this state. Cells were fixed in methanol at different time points following release from depolymerizing conditions, and host microtubules were stained via indirect immunofluorescence for tubulin and EB1 (end-binding protein 1) using antibodies from Santa Cruz Biotechnology (Dallas, TX, USA These studies also attested to the reactivity and specificity of both antibodies.

## Data Availability

All data from this research are included in this paper.
